# Input-Output Budget of Nitrogen and the Effect of Experimentally Changed Deposition in the Forest Ecosystems in Central Japan

**DOI:** 10.1100/tsw.2001.281

**Published:** 2001-11-08

**Authors:** Junko Shindo, Tamon Fumoto, Noriko Oura, Hideshige Toda, Hiroyuki Kawashima

**Affiliations:** National Institute for Agro-Environmental Sciences, Tsukuba, Ibakari, Japan

**Keywords:** nitrogen deposition, nitrogen saturation, Japanese terrestrial ecosystems, material cycle

## Abstract

To evaluate the current nitrogen (N) status in Japanese forests, field measurements of rainfall, throughfall, litter layer percolation, and soil solution percolation were conducted in a red pine stand (Kannondai) and a deciduous stand (Yasato) located in central Japan. N input via throughfall was 31 and 14 kg ha yearand output below rooting zone was 9.6 and 5.5 kg ha year in Kannondai and in Yasato, respectively. Two thirds of input N were retained in plant-soil systems. Manipulation of N input was carried out. Ionic constituents were removed from throughfall with ion exchange resin at removal sites and ammonium nitrate containing twice the N of the throughfall was applied at N addition sites periodically. SO_4_ output below 20-cm soil layer changed depending on the input, while NO output was regulated mainly by the internal cycle and effect of manipulation was undetected. These Japanese stands were generally considered to have a larger capacity to assimilate N than NITREX sites in Europe. However, N output fluxes had large spatial variability and some sites in Kannondai showed high N leaching below rooting zone almost balanced with the input via throughfall.

## INTRODUCTION

Human activity has converted the atmospheric nitrogen molecules ($N_2$) to reactive nitrogen (NOy, NHx, and organic N). Galloway et al.[1] estimated that the rate of anthropogenic N fixation was now similar to the natural biological fixation rate; about 78 Tg N year$^{-1}$ by fertilizer production, 43 Tg N year$^{-1}$ by legume production, and 21 Tg N year$^{-1}$ by energy production (combustion of fossil fuel). Atmospheric deposition of N compounds of anthropogenic origin has caused the changes in nutrient cycling in forest ecosystems and has led to N saturation in Europe and eastern North America[2,3]. Intensive studies have been conducted in these regions on the relation between $NO_3^-$ leaching to groundwater and N status of ecosystems, possible effects of long-term enhanced N deposition on N status, and recovery processes of N saturation to N-limited status, etc.[4]

Currently, East Asia has received attention in terms of N mobilization[5]. Its growing population and rapid economic development have markedly enhanced the emission of reactive N in the latest 2 decades. We do not have sufficient data, however, to evaluate the N deposition rates in East Asia. According to the national monitoring of acidic deposition in Japan during 1993 to 1997, yearly wet deposition rates of N at 44 sites throughout Japan ranged from 2.4 to 12.5 kg N ha$^{-1}$ year$^{-1}$ (6.8 on average)[6]. Fujita et al.[7] estimated that wet deposition rate of N was 547 Gg N year$^{-1}$ ($NH_4^+$:336 and $NO_3^-$:211) in Japan, approximately corresponding to 7.5 kg N ha$^{-1}$ year$^{-1}$, based on the data at

21 sites representing each climatic region. Domestic N emission was estimated to be 810 Gg N year$^{-1}$, and the difference between emission and wet deposition was mainly attributed to the dry deposition[7]. Dry deposition data used to evaluate global distribution have not been available for Japan and for East Asia.

N saturation has been recently reported in Japanese forests[8,9]. However, N status and processes of N saturation caused by the excess N in the Asian ecosystems including Japan have not been well-understood, where meteorological condition, soil, vegetation, etc., are different from those in Europe and North America. The major aim of this study is to evaluate the current N status in Japanese forests located at the low-to-medium elevation in central Japan based on the field measurements and the N input manipulation experiments.

## METHOD AND MATERIALS

### Field Measurements

We conducted field measurements at two forest stands: Kannondai and Yasato in Ibaraki Prefecture in central Japan. Kannondai stand (140°6′33″E, 36°1′22″N) is 20 m above sea level and is covered with 30-year-old Japanese red pine (*Pinus densiflora*) growing on volcanic ash soil. Yasato stand is on top of a hill (an elevation of 380 m) located 30 km north of Kannondai, covered with deciduous mixed trees. Main tree species are *Quercus serrata*, *Prunus jamasakura*, *Rhododendron obtusum*, etc. Soil of Yasato stand is brown forest soil originated from granitic materials affected by the volcanic ash[10].

Rainfall, throughfall, litter layer percolation, and soil solution at several depths were collected periodically: every 7 to 10 days in Kannondai, every month in Yasato during 1997 and 1988, and every 2 weeks in both stands since 1999. Sampling point numbers and measurement starting dates are shown in Table 1. Rainfall and throughfall water were collected with a funnel of 20-cm diameter and stored in a polyethylene bottle through 8-mm membrane filter. Forest floor (litter layer) was removed and put on the Büchner funnel of 13-cm diameter and placed at the ground level. Litter layer percolation was collected in a polyethylene bottle under the funnel. Ceramic tension lysimeters were installed in the soil at several depths, and soil solution was collected by reducing the pressure of the connected bottle from –55 to –60 cm Hg for 1 to 2 days. The collected samples were filtered with a 0.22-mm membrane filter, and the concentrations of $Na^+$, $NH_4^+$, $K^+$, $Mg^{2+}$, $Ca^{2+}$, $Cl^-$, $NO_3^-$, and $SO_4^{2-}$ were measured by ion chromatography (Dionex DX500).

**TABLE 1**
**Overview of the Field Measurements and N Input Manipulation Experiment in Each Stand**

| | | Replication | | Start From | | Manipulation of N Input |
|---|---|---|---|---|---|---|
| Rainfall | | 2 | | Sep–97 | | |
| Throughfall | | 4 | 2 | Sep–97 | | |
| | | | 2 | Jun–99 | | |
| Litter layer percolation | | 19 | 3 | Sep–97 | | No treatments |
| | | | 12 | Jun–99 | 4 | N remove |
| | | | | | 4 | No treatments |
| | | | | | 4 | N add |
| | | | 4 | Mar–00 | | No treatments |
| Soil water | 20-cm depth | 9 | 3 | Sep–97 | | No treatments |
| | | | 6 | Jun–99 | 2 | N remove |
| | | | | | 2 | No treatments |
| | | | | | 2 | N add |
| | 40-cm depth | 6 | | Jun–99 | 2 | N remove |
| | | | | | 2 | No treatments |
| | | | | | 2 | N add |
| | 50-cm depth | 3 | | Sep–97 | | No treatments |
| | 60-cm depth | 6 | | Jun–99 | | Same as the 40 cm depth |
| | 100-cm depth | 6 | | Jun–99 | | Same as the 40 cm depth |

We measured the hourly amount of precipitation with a rain gauge and hourly volumetric soil moisture contents of 0- to 20- and 20- to 50-cm depths with time domain reflectometry (TDR). These data were compiled into daily values and used to estimate the vertical water flux in the soil under the following assumptions:

1. Daily water percolation from the litter layer is proportional to the daily precipitation.
2. Soil water percolates downward only when soil moisture content is larger than the field capacity.
3. The field capacity is estimated as the average moisture contents of the second day after heavy rain more than 20 mm during October to next May (excluding summer season).

The daily vertical water flux, $W_{flux}(t)$ (cm day$^{-1}$) on the day t was calculated with the equation:

$$W_{flux}(t) = \max(W(t-1) + W_{in}(t) - \max(W(t), W_f), 0)$$

where W(t)(cm) is water content derived from the daily water moisture content multiplied with the soil thickness of the objective soil layer, $W_{in}(t)$ (cm day$^{-1}$) is amount of infiltrating water into the soil layer from the litter layer or from the upper soil layer, and $W_f$ (cm) indicates the field capacity. We estimated the water percolation rate from 20-cm depth of soil and from 50-cm (40- to 60-cm) depth according to the above equation. Water flux at the depth of 100 cm was assumed equal to the value at 50-cm depth. Based on these water flux estimation and element concentration in soil solution, element fluxes from several depths of the surface soil and below the rooting zone (below 100 cm) were calculated.

## N Manipulation Experiments

To investigate the effect of N deposition change on the N dynamics in the plant-soil system, we carried out the field experiments manipulating the N input as shown in Table 1. For the N removal, two sets of roofs were installed under the canopy to gather the throughfall in each stand. A roof of 1 × 0.5 m was formed by 8 connected square funnel of 25 × 25 cm made from transparent PVC as shown in Fig. 1. Throughfall water was filtered by glass wool and 8-mm membrane filter and went into the columns connected in series. The upper column contained about 25 ml of anion exchange resin (Bio-Rad AG1-X8), and the lower column contained the same amount of cation exchange resin (AG500W-X8). The water having ionic constituents removed fell through eight needles onto the forest floor. The resin columns were replaced with the new ones every several months. A funnel of 25 × 25 cm of the same structure (without needles) as the removal roof was put beside the roof, and all water fallen through the column was collected and analyzed in the concentrations of ionic species to evaluate each ion's removal ratio. These values were used to signal that the resin column should be replaced to the new ones. Amounts of base cations ($K^+$, $Mg^{2+}$, and $Ca^{2+}$) leached from canopy for a roof area (0.5 m$^2$) were evaluated based on the assumption that increase in $Na^+$ in throughfall was all due to dry deposition and that the dry deposition composition was identical to that of wet deposition. A 2-l aqueous solution, which contained corresponding amount of KCl, $MgCl_2$, and $CaCl_2$ to the canopy leaching, was added to the forest floor of the roof area after collecting samples to compensate the root uptake for canopy leaching. Under the roof, we collected litter layer percolation (two replications) and soil solutions with four tension lysimeters at 20-, 40-, 60-, and 100-cm depth, respectively. Square funnels of 25 × 25 cm were installed for collection of the litter layer percolation instead of Büchner funnels of 13-cm diameter. An open top/bottom chamber of 50 × 50 cm was placed under the roof, and lysimeters were installed inside the chamber. The height of the chamber was 20 cm in Kannondai and 40 cm in Yasato, and the bottom half of that was buried in the soil to prevent the lateral water flow on the forest floor and in the surface soil layer. The ceramic tension lysimeter installed in June 1999 was placed in such a chamber. These chambers were used to measure the emission rate of $N_2O$ from forest floor[11], though we do not describe the $N_2O$ emission in this paper.

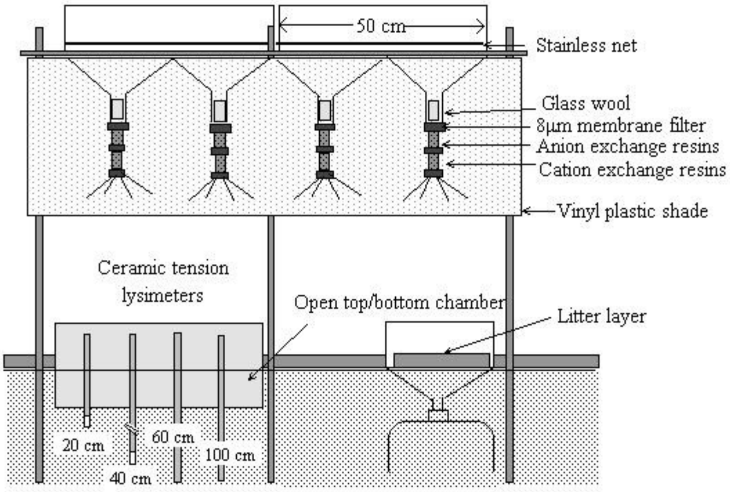

Figure 1 The nitrogen removal roof and the sampling system

**FIGURE 1**. The nitrogen removal roof and the sampling system.

Two N addition sites of 0.5 $m^2$ were assigned in each stand. For the N addition, 2 l of $NH_4NO_3$ solution, which contained twice as much N in throughfall, was applied to the 0.5-$m^2$ area where a chamber with lysimeters and two funnels with litter layer were installed. N addition was done every 2 weeks, immediately after the regular sampling.

We started removal manipulation in January 2000 in Kannondai, in February 2000 in Yasato, and N addition in late May 2000 in both stands.

## RESULT AND DISCUSSION

### Yearly Flux of N and Other Ions

Table 2 shows soil properties of each stand and yearly N deposition from June 1999 and June 2000. Yasato soil was more acidic and its base saturation (BS) was about 5% both in surface soil and in subsoil. BS values in Kannondai soil were 13 and 24%, in surface and subsoil, respectively. N deposition rates changed annually, but we could not find increasing or decreasing trends in the data when we compared the values from 1997 to 2001. About 66% (63 to 67%) of N in rain and 50% (35 to 57 %) of N in throughfall were $NH_4^+$ in both stands.

Fig. 2 shows yearly fluxes of ionic constituents from rainfall to soil water percolation from 100-cm depth, calculated based on the data from June 1999 to May 2001 at the sites without N manipulation. Table 3 also indicates the yearly N fluxes for the same period at the same sites. For the base cations ($K^+$, $Mg^{2+}$, and $Ca^{2+}$) patterns of ion flux from rain to percolation from 100-cm depth were similar to each other in Kannondai and in Yasato. For other ions, inputs via throughfall and litter layer percolation were larger in Kannondai than in Yasato, which was attributed to larger contribution of dry deposition of the coniferous trees in Kannondai. It was observed that $NH_4^+$ was retained and assimilated in the canopy in Yasato. Biological activities were high in soil system in both stands. Nitrification and preferable uptake of ammonium ion were quite active in the topsoil layer. $NO_3^-$ was kept at high concentrations in the upper soil layer until 40- to 60-cm depth, and below that it was consumed for assimilation and denitrification. Mineralization of organic matter may

**TABLE 2**
**Soil Properties, Annual Precipitation, and N Deposition Rates**

| | | Kannondai (Red Pine) | | Yasato (Mixed Deciduous) | |
|---|---|---|---|---|---|
| C/N in litter layer | | 36.9 | | 25.2 | |
| Soil pH ($H_2O$) | 0–20 cm | 5.5 | | 4.9 | |
| | 20–50 cm | 6.0 | | 5.3 | |
| Soil C:N ratio | 0–20 cm | 16.4 | | 16.0 | |
| | 20–50 cm | 13.4 | | 15.5 | |
| | | June 1999–May 2000 | June 2000–May 2001 | June 1999–May 2000 | June 2000–May 2001 |
| Annual rainfall (mm) | | 1169 | 1499 | 1137 | 1701 |
| N deposition (kg N $ha^{-1}$ $year^{-1}$) | Rainfall | 13.9 | 22.0 | 16.0 | 14.1 |
| | Throughfall | 32.9 | 31.9 | 12.1 | 14.3 |

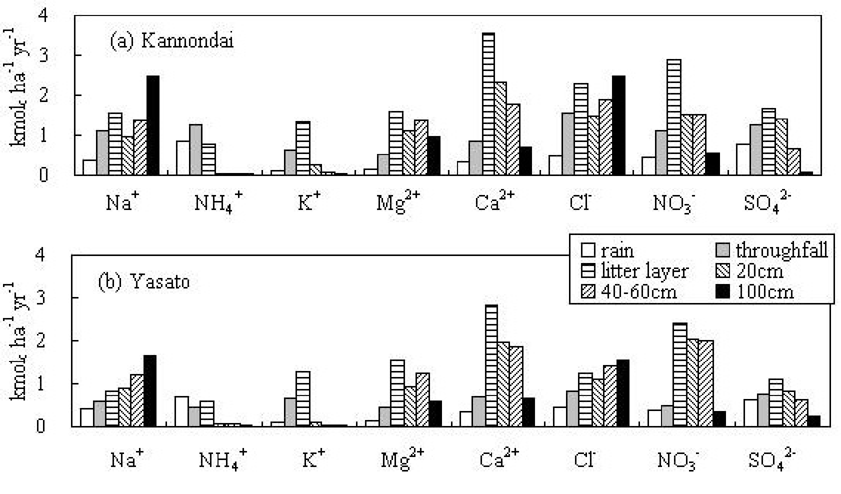

**FIGURE 2**. Yearly fluxes of ionic constituents in the area without nitrogen manipulation (June 1999 to May 2001).

**TABLE 3**
**Average Fluxes of N in the Area without N Manipulation (kg N ha$^{-1}$ year$^{-1}$)**

| | Rain | Throughfall | Litter Layer | 20 cm | 40–60 cm | 100 cm |
|---|---|---|---|---|---|---|
| Kannondai | 18.2 | 32.9 | 51.1 | 21.9 | 21.7 | 9.63* |
| Yasato | 15.0 | 13.1 | 42.0 | 29.2 | 28.8 | 5.5 |

* Average of two sites whose values were very different from each other (1.5 and 17.9 kg N ha$^{-1}$ year$^{-1}$)

contribute to the high $NO_3^-$ concentration in upper soil layer. These processes in soil layers seemed more active in Yasato stand than in Kannondai.

Amount of N outputs below the rooting zones at the both stands were intermediate compared with the NITREX sites[12]. Outputs were much smaller than the inputs via throughfall in both Japanese stands. $NO_3^-$ concentrations in groundwater in Kannondai were also quite low, ranging from 0.001 to 0.032 mmol$_c$ l$^{-1}$ (mean 0.008 mmol$_c$ l$^{-1}$). Though the N input in Kannondai, 32.9 kg N ha$^{-1}$ year$^{-1}$ was smaller than the values at Dutch sites being N saturated, it was substantially large and close to the value in Solling (36.5 kg N ha$^{-1}$ year$^{-1}$) where output flux of N was 22 kg N ha$^{-1}$ year$^{-1}$. The large C:N ratio of forest floor in Kannondai shown in Table 2 might explain the relatively small output N flux for the large input[13].

The sulfate budgets in these Japanese stands were quite different from those at the NITREX sites. Most of the sulfate infiltrated into soil layer was retained in the soil-plant system and output flux was small in the Japanese stands in contrast to the NITREX sites where $SO_4^{2-}$ input and output was generally balanced[12]. It was considered that soil minerals originating from volcanic ash at the Japanese stands worked to absorb the $SO_4^{2-}$. Most of the Japanese soils have been partly affected by volcanic ash even when the soils are not classified as volcanic ash soil, like Yasato stand [10].

## Effect of N Manipulation

Removal ratios of ions by ion exchange resin were 55 to 92% for cations and 92 to 97% for anions. Average pH of the water coming through the resin column was 5.4. Resin could effectively remove anions, and N input reduced to 4.7 kg ha$^{-1}$ year$^{-1}$ and 1.5 kg N ha$^{-1}$ year$^{-1}$ in Kannondai and in Yasato, respectively, though forest floor under the roof was considered to receive more N, because the throughfall water falling around it partly came in. At the N addition sites, yearly N input after manipulation (from June 2000 to May 2001) resulted in 101 kg and 43 kg N ha$^{-1}$ year$^{-1}$ in Kannondai and in Yasato respectively.

Ionic concentrations of litter layer percolation and soil solution varied markedly with time and by sampling sites even without N input manipulation. Fig. 3 shows the 2-year temporal changes in $NO_3^-$ concentration of soil solution at 20-cm depth at the five sites without any N manipulation in Kannondai. Temporal changes were especially large in two sites, indicated as P1 and P2. A 2-year average concentration of each sampling site had a large variety, ranging from 0.12 to 0.38 mmol$_c$ l$^{-1}$. Therefore direct comparisons of the N concentration between sites with different N manipulation were unsuitable evaluations of the effect of manipulation.

We calculated the differences in the average daily element fluxes between the period of premanipulation (before removal) and postmanipulation (after N addition), and evaluated the effects of manipulation on these differences. Fig. 4 shows the daily ion fluxes from litter layer and surface soil layer (20-cm depth) in Kannondai for $N_4^+$, $NO_3^-$, and $SO_4^{2-}$ ions. In respect to the litter layer percolation, average daily fluxes in the premanipulation period were smaller than the values in the postmanipulation period except for ammonium ion flux at the removal sites. These increasing amounts for each ion were growing in the order of the removal, the nonmanipulation and the addition, and the effects of the manipulation were highly significant with 99% confidence limits. For $SO_4^{2-}$ fluxes, data from nonmanipulation sites and N addition sites were merged and compared with the removal sites' data, as $SO_4^{2-}$ ion was not added at the N addition sites.

In case of the soil solution percolation, the relation between manipulation and daily flux changes was unclear. Increasing amounts of $SO_4^{2-}$ fluxes were related to the manipulation with the 90% confidence level. For N fluxes, both $N_4^+$ and $NO_3^-$, however, no effects of N addition of 100 kg ha$^{-1}$ year$^{-1}$ could be found,

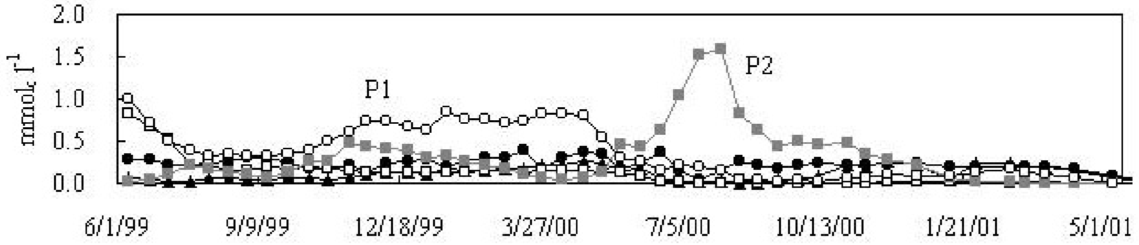

**FIGURE 3.** Temporal changes in nitrate concentration of soil solution at 20-cm depth at the no-manipulation areas in Kannondai.

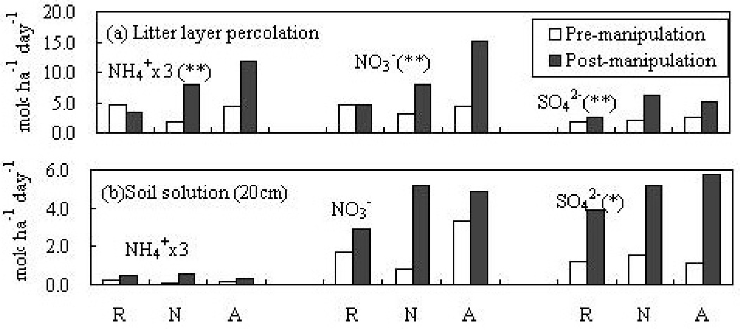

**FIGURE 4**. The effects of N manipulation to the daily ion flux from (a) litter layer and (b) soil solution (20-cm depth) in Kannondai. Confidende limit: 99% (**), 90% (*), R: removal, N: no manipulation, A: addition.

though the increasing amounts at the removal sites tended to be lower than those at the nonmanipulation sites (Fig. 4b). In Yasato, the deciduous stand, we could not detect the effect of N manipulation on the $N_4^+$ flux even for the litter layer percolation. Changes in flux from 20-cm soil corresponding to the manipulation was only observed qualitatively for the $SO_4^{2-}$ flux as shown in Fig. 5 (statistically insignificant). No manipulation effects to the deeper soil solution were observed either in Kannondai or in Yasato. N output in soil leachate at 100 cm was quite stable at the sites of both stands despite the large changes in N input, though output fluxes were intermediate. The exceptions were P1 site (a nonmanipulation site) shown in Fig. 3 and adjacent N-removal site in Kannondai. At these sites, $NO_3^-$ concentration at 100-cm depth raised steadily from 0.02 to 0.31 $mmol_c\ l^{-1}$ in 2 years, synchronizing to the decrease in the concentration at 20-cm depth shown in Fig 3. Yearly N output for these two sites corresponded to about 30 kg $ha^{-1}$ $year^{-1}$ in the second year (June 2000 to May 2001). The high $NO_3^-$ discharge at these sites was attributed to local disturbance of the internal soil cycle. These data show that N status varied markedly in a narrow space and some N-rich spots might exist locally.

According to the NITREX experiments, experimentally changed N deposition caused rapid change in the output flux of $NO_3^-$ in European ecosystems[14]. Among the six sites where N addition experiments were conducted, four sites showed marked increase in N output in soil leachate at over 50-cm depth or runoff in a year. Three of them became N-saturated status, where N output was balanced with or exceeded the input, in 2 years. N outputs were dramatically reduced by the N removal experiment in the first year at the N-saturated sites. On the contrary, N concentration changes in soil solution seemed to be regulated by the internal circulation from biological and chemical processes in the plant-soil systems, not by the external input, in these Japanese ecosystems. As $SO_4^{2-}$ is less involved in biological cycles than N, the external $SO_4^{2-}$ input had the relatively larger contribution to the soil solution chemistry, which reacted more promptly to the input change of $SO_4^{2-}$ than of N.

These Japanese ecosystems seem to have larger capacity to accept N than the ecosystems in North America and Europe. Plants and microorganisms probably had higher activities to assimilate N and to denitrify due to higher temperature and higher precipitation and increasing N deposition might stimulate these activities. Studies on base cation budget and strontium isotope ratio indicated that base cation supplies from soil were relatively high in these stands: 2.08 $kmol_c$ $ha^{-1}$ $year^{-1}$ ($Ca^{2+}$), 0.86 ($Mg^{2+}$) in Kannondai, 1.35 ($Ca^{2+}$), and 0.89 ($Mg^{2+}$) in Yasato for surface soils of 0.5-m thickness[10]. It was considered that the enhanced assimilation still did not bring about the nutrient imbalance be-

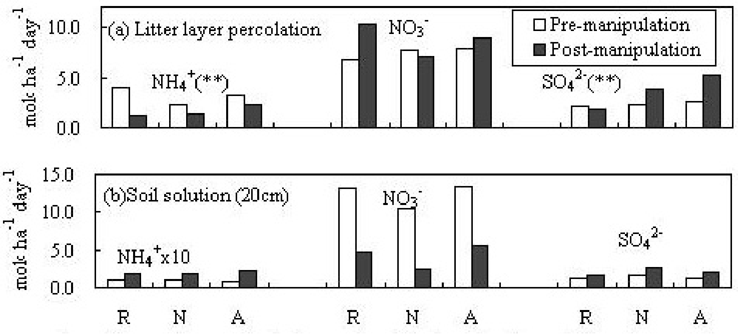

**FIGURE 5.** The effects of N manipulation to the daily ion flux from (a) litter layer and (b) soil solution (20-cm depth) in Yasato. Confidende limit: 99% (**), 90% (*), R: removal, N: no manipulation, A: addition.

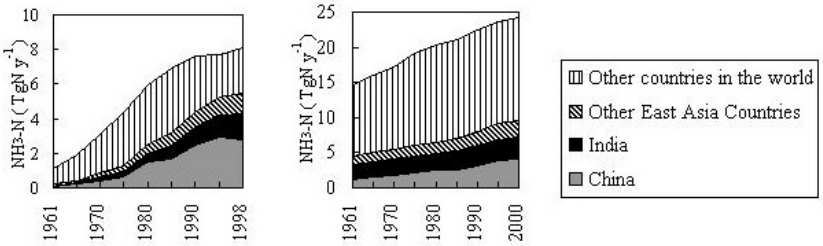

**FIGURE 6.** Yearly changes in $NH_3$ emission from (a) fertilized soil and (b) livestock excreta.

cause of the high ability of base cation supply of the soils. Ohrui et al.[8] and Mitchell et al.[9] suggested, however, that such conditions (warm temperatures and abundant moisture) caused the high rates of N mineralization and induced N leaching in some Japanese ecosystems. Further study will be necessary to derive the relation between N retention and N mineralization in the warm, moist, and N-rich conditions in Asia including Japan and clarify the factors regulating these processes.

## CONCLUSIONS

Field measurements and N-input manipulation conducted in two forests in central Japan demonstrated that these ecosystems responded to changing N input quite differently from the European ecosystems investigated by the NITREX project. In these Japanese stands, large amounts of N were cycling internally in the plant-soil system, and external N input had little effect on the internal cycle. Despite the high N input, two thirds of added N was retained in the system to be assimilated by plants and microorganisms or be denitrified. A warm climate, high precipitation, and the relatively large weathering rate of the soil contributed to enhanced assimilation. However, some N-rich spots were identified in the area where N output was almost balanced with the input.

Agriculture in East Asia (up to Pakistan) is now emitting a considerable amount of $NH_3$. East Asian contribution to $NH_3$ emission from fertilized soils and livestock excreta are about 70% and 40%, respectively, of the world. They are still increasing (Fig. 6), according to the FAO statistical data[15] on fertilizer consumption and number of heads of livestock and emission factors derived by Matthews[16] and Bouwman et al.[17]. Moreover, Van Aardenne et al.[18] estimated that $NO_x$ emission in the same region will increase from 5.83 Tg N $year^{-1}$ in 1990 to 26.2 Tg N $year^{-1}$ in 2020. Intensive study is necessary to understand the current N status and effect of these increasing amounts of N input on it in Asian ecosystems.

## References

1. Galloway, J.N., Schlesinger, W.H., Levy, H.,II, Michaels, A., and Schnoor, J.L. (1995) Nitrogen fixation: anthropogenic enhancement-environmental response. *Glob. Biogeochem. Cy.* **9**, 235–252.
2. Aber, J.D., Nadelhoffer, K.J., Steudler, P, and Melillo, J.M. (1989) Nitrogen saturation in northern forest ecosystems; excess nitrogen from fossil fuel combustion may stress the biosphere. *Bioscience* **39**, 378–386.
3. Wright, R.F., Roelofs, J.G.M., Bredemeier, M., Blanck, K., Boxman, A.W., Emmett, B.A., Gundersen, P., Hultberg, H., Kjønaas, O.J., Moldan, F., Tietema. A, and Van Breemen, N. (1995) NITREX: responses of coniferous forest ecosystems to experimentally changed deposition of nitrogen. *For. Ecol. Manage.* **71**, 163–169.
4. Tietema, A., Boxman, A.W., Bredemeier, M., Emmett, B.A., Moldan, P., Gundersen, P., and Wright, R.F. (1998) Nitrogen saturation experiments (NITREX) in coniferous forest ecosystems in Europe: a summary of results, In *Proceedings of the 1st International Nitrogen Conference.* van der Hoek, K.W., et al. Eds. Elsevier, New York. pp. 433–437.
5. Galloway, J.N. (2000) Nitrogen mobilization in Asia. *Nutr. Cy. Agroecosyst.* **57**, 1–12.
6. Anon. (1999) Data Report of The Third Survey on Acid Deposition. Japan Environmental Agency (CD-ROM by Acid Deposition and Oxidant Research Center).
7. Fujita, S., Takahashi, A., Hayami, H., and Sakurai, T. (2000) Wet deposition of nitrate and ammonium over the Japanese Archipelago. *Environ. Sci.* **13**, 491–501 (in Japanese).
8. Ohrui, K. and Mitchell, M.J. (1997) Nitrogen saturation in Japanese forested watersheds. *Ecol. Appl.* **7**, 391–401.
9. Mitchell, M.J., Iwatsubo, G., Ohrui, K., and Nakagawa, Y. (1997) Nitrogen saturation in Japanese forests: an evaluation. *For. Ecol. Manage.* **97**, 39–51.
10. Shindo, J., Fumoto, T., Oura, N., Nakano, T., and Takamatsu, T. (2001) Estimation of mineral weathering rates under field conditions based on base cation budget and strontium isotope ratios. *Water Air Soil Pollut.* in press.
11. Oura, N., Shindo, J., Fumoto, T., Toda, H., and Kawashima, H. (2001) Effects of nitrogen deposition on nitrous oxide emissions from the forest floor. *Water Air Soil Pollut.* in press.
12. Bredemeier, M., Blanck, K., Xu, Y.-J., Tietema, A., Boxman, A.W., Emmett, B., Moldan, F., Gundersen, P., Schleppi, P., and Wright, R.F. (1998) Input-output budgets at the NITREX sites. *For. Ecol. Manage.* **101**, 57–64.
13. Gundersen, P., Callesen, I., and de Vries, W. (1998) Nitrate leaching in forest ecosystems is related to forest floor C/N ratios. In *Proceedings of the 1st International Nitrogen Conference*. van der Hoek, K.W., et al. Eds. Elsevier, New York. pp. 403–407.
14. Wright, R.F., Roelofs, J.G.M., Bredemeier, M., Blanck, K., Boxman, A.W., Emmett, B.A., Gundersen, P., Hultberg, H., Kjonaas, O.J., Moldan, F., Tietema, A., van Breemen, N., and van Dijk, H.F.G. (1995) NITREX: responses of coniferous forest ecosystems to experimentally changed deposition of nitrogen. *For. Ecol. Manage.* **71**, 163–169.

15. Anon. (2000) Statistical database. The Food and Agriculture Organization of the United Nations. Rome. http://apps.fao.org/c/s.dll/nph-db.pl
16. Matthews, E. (1994) Nitrogenous fertilizers: global distribution of consumption and assciated emissions of nitrous oxide and ammonia. *Glob. Biogeochem. Cy.* **8**, 411–439.
17. Bouwman, A.F., Lee, D.S., Asman, W.A.H., Dentener, F.J., van der Hoek, K.W., and Olivier, J.G.J. (1997) A global high-resolution emission inventory for ammonia, *Glob. Biogeochem. Cy.* **11**, 561–587.
18. Van Aardenne, J.A., Carmichael, G.R., Levy, H. II, Streets, D., and Hordijk, L. (1999) Anthropogenic $NO_x$ emissions in Asia in the period 1990–2020. *Atmos. Environ.* **33**, 633–646.

| | | |
|---|---|---|
| Received: | August | 9, 2001 |
| Revised: | September | 30, 2001 |
| Accepted: | October | 5, 2001 |
| Published: | November | 8, 2001 |
